# Does Coffee Have Terroir and How Should It Be Assessed?

**DOI:** 10.3390/foods11131907

**Published:** 2022-06-27

**Authors:** Simon D. Williams, Bronwyn J. Barkla, Terry J. Rose, Lei Liu

**Affiliations:** Southern Cross Plant Sciences, Faculty of Science and Engineering, Southern Cross University, Lismore, NSW 2480, Australia; simon.williams@scu.edu.au (S.D.W.); bronwyn.barkla@scu.edu.au (B.J.B.); terry.rose@scu.edu.au (T.J.R.)

**Keywords:** sensory experience, environment, post-harvest, maturation, roasting, particle size

## Abstract

The terroir of coffee is defined as the unique sensory experience derived from a single origin roasted coffee that embodies its source. Environmental conditions such as temperature, altitude, shade cover, rainfall, and agronomy are considered the major parameters that define coffee terroir. However, many other parameters such as post-harvest processing, roasting, grinding, and brewing can combine to influence the perception of terroir. In this review, we discuss the contribution of these parameters and their influence on coffee terroir. Assessment of terroir requires defined sensory descriptors, as provided by the World Coffee Research Lexicon, and standardized roast level, grind size, and brew method. The choice of the post-harvest processing method is often environmentally dependent, suggesting that an inclusion into the coffee terroir definition is warranted. Coffee terroir is often not intentionally created but results from the contributions of the *Coffea* species and variety planted, environmental and agricultural parameters, and both the harvest and post-harvest method used. The unique combination of these parameters gives the consumer a unique cup of coffee, reminiscent of the place the coffee was produced.

## 1. Introduction

Terroir is the complex interaction of environmental, varietal, and agricultural factors that affect a product’s sensory experience [1]. The terroir of wine is widely recognized, with the vineyard location considered to affect a wine’s sensory qualities [2]. Plantings of clonal wine grapes exhibit chemical fingerprint variation dependent on the vineyard location, supporting terroir variation [3]. Factors such as air temperature, solar radiation, rainfall, soil water holding capacity, and nitrogen are associated with locations and significantly contribute to the terroir of wine [4].

Terroir in coffee is most notable in single-origin coffees that represent their growing location [5]. Like the terroir of wine, the terroir of coffee is affected by environmental factors such as latitude, longitude, rainfall, temperature, and altitude [1,5,6]. It has been reported that terroir associated with high altitude (>1000 m) and low rainfall (<1600 mm/year) provided a more appreciated coffee that was aromatic, slightly bitter, acidic, and had body [1]. In contrast, coffee grown at low altitude (<850 m) and high rainfall (>2110 mm/year) was less appreciated, with more substantial bitterness, grassy flavor, lower aroma, and increased astringency [1]. A terroir that includes high altitude is reported to produce a favorable, higher quality coffee [1,6]. It is hypothesized that higher altitude with a lower average temperature prolongs the coffee cherry maturation period, resulting in a nutritionally dense coffee bean that provides a greater concentration of chemicals/flavor to the resulting coffee beverage [1,5,6].

The wine industry [7] has defined and created a formal assessment of wine terroir, involving a panel of wine experts that compares a wine sensory experience to a benchmark [8]. In comparison, coffee terroir currently does not have a formal definition. Current assessment of the terroir of roasted coffee involves either the use of a scoring system developed by the specialty coffee association (SCA) [9] or the use of sensory descriptions with references made to growing locations [5].

**Figure 1:**
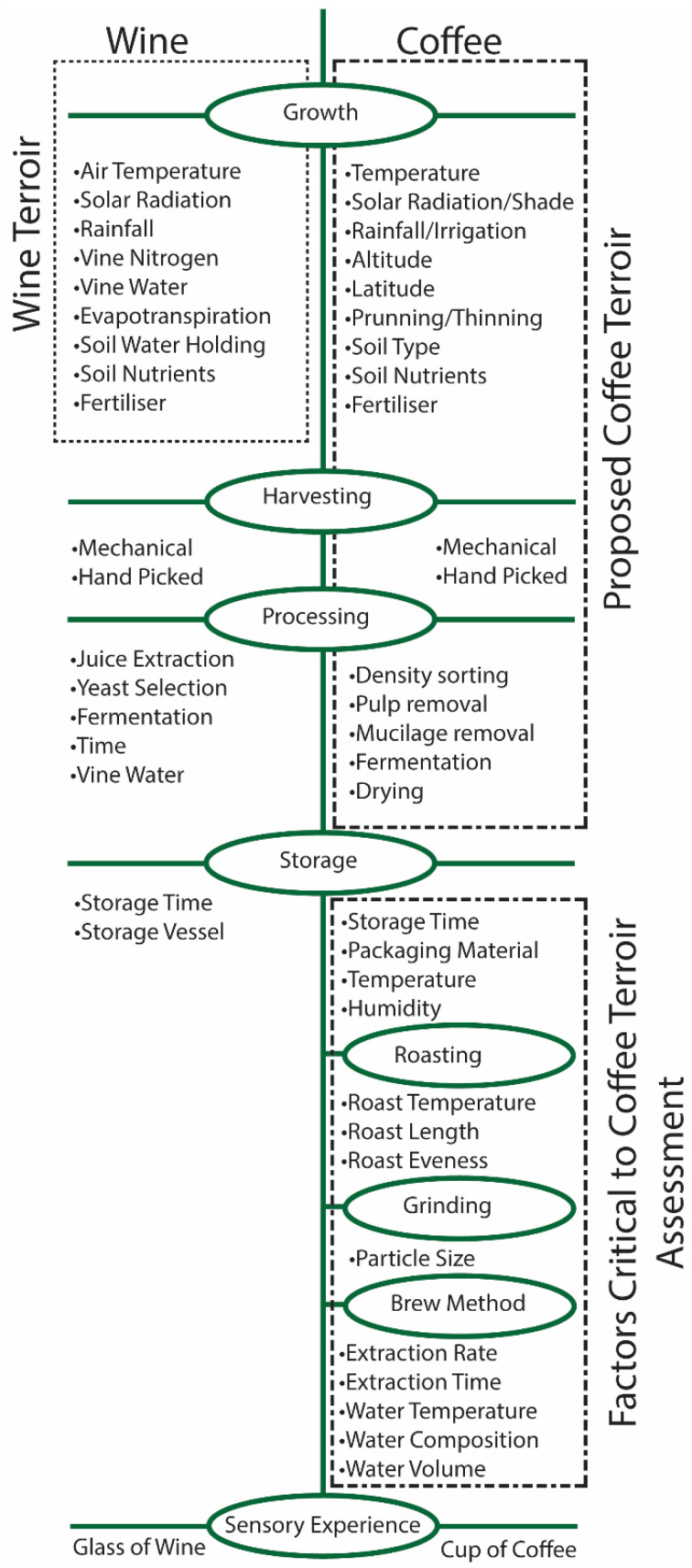
A comparison of the steps required before the terroir of wine or coffee is assessed, with wine terroir defined [7] and coffee terroir proposed.

Based on the terroir definition of wine, terroir is fixed once the coffee cherry is harvested (Figure 1). However, compared to wine, coffee undergoes more steps in its production pathway that can affect the final sensory experience (Figure 1). The impact of the processing method, roast level, and brew method on the final sensory experience is well documented and has been recently reviewed [10,11,12,13,14]. As these steps (Figure 1) affect the final sensory experience, they significantly affect the terroir experience and assessment. The observation of coffee terroir is possible when the roasting, grinding, and brewing steps are standardized [15].

Further, compared to wine, the post-harvest processing of coffee often occurs under uncontrolled environmental conditions. Sundried and monsoon Malabar represent post-harvest processing methods that are particularly reliant on the environment [10,14]. As the environment still affects the processing steps, consideration should be given to whether coffee terroir encompasses this step (Figure 1).

Coffee terroir broadly encompasses those factors that affect the growth of the coffee cherry and its conversion to a green coffee bean [16]. However, the assessment of coffee terroir depends on the steps that convert the green coffee bean into a cup of coffee (Figure 1) [10]. This review aims to identify and bring together the factors determining the terroir and the factors affecting the terroir assessment. The identification of terroir begins with the quality of the cup of coffee. This review works from the cup to the farm to assess the factors that influence the terroir of coffee.

## 2. Factors Affecting the Terroir Assessment

### 2.1. Coffee Sensory Experience and Analysis

The coffee industry uses three general methods for sensory analysis: a triangle method for distinguishing between two coffees, a scoring method focused on ranking key/overall sensory attributes in a cup of coffee, and a descriptive method that provides more in-depth details of the sensory descriptors [17]. These methods allow for the assessment and comparison of the coffee sensory experience with minimal bias from the taster.

The triangle method requires the preparation of three cups of coffee, one of which is different. The taster then determines the deviating cup. The triangle test is primarily used by the industry for quality control and matching coffee blends [17].

**Figure 2:**
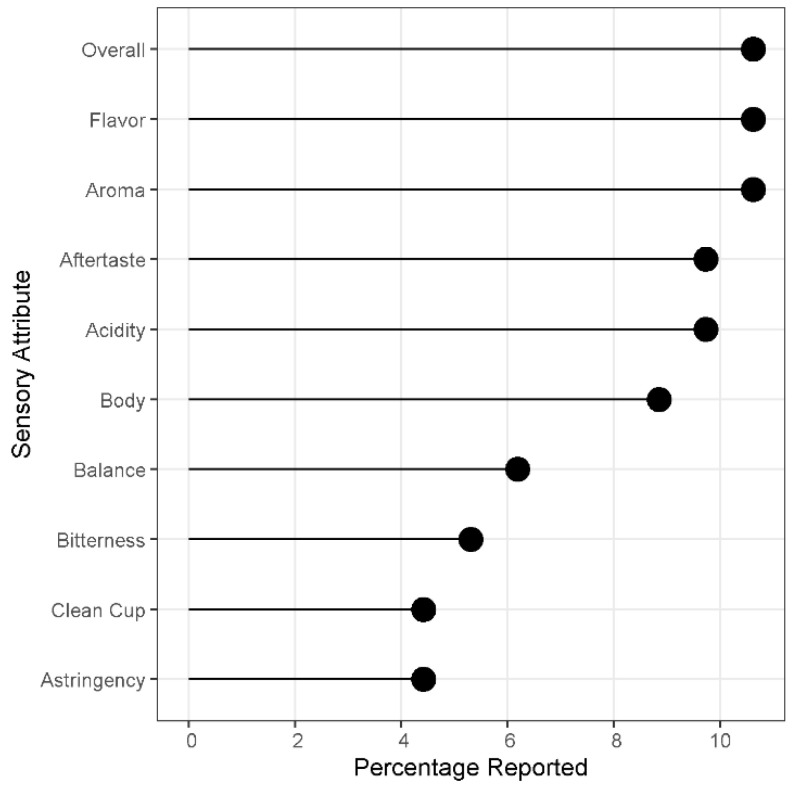
The top eleven sensory attributes used for assessing overall sensory quality across the reviewed literature. Percentage reported is the number of occurrences of an attribute out of the total number of reported attributes [1,21,22,23,24,25,26,27,28,29,30,31,32,33,34,35,36,37,38,39,40].

The overall scoring method uses industry-standard guidelines to assess and score coffee quality [18]. Evaluating the aroma, flavor, aftertaste, acidity, body, balance, sweetness, cleanness, uniformity, and overall impression of the coffee via a scoring system provides a coffee quality score [18]. The score allows coffees to be ranked and statistics to be more readily applied. Industry uses the scoring system to assign value, with higher scoring coffee receiving higher prices [9]. Overall impression, flavor, aroma, aftertaste, acidity, and body are the most frequently published attributes (Figure 2).

The descriptive sensory analysis provides a more in-depth study of the sensory attributes [19]. The World Coffee Research Sensory Lexicon defines 110 sensory descriptors related to coffee [20]. For analysis, 110 descriptors is an impractical number to assess, requiring a subset as a compromise between the number of attributes for assessment and the time allowed to assess a sample [21].

The scoring and descriptive sensory analysis methods are capable of defining terroir. The SCA overall scoring method combined with ad hoc sensory descriptors such as sweet, complex, fruit, floral, berry, caramel, butter, cherry, wine, peach, and juicy identified country-level terroir in Central America, with coffee from Guatemala receiving higher scores [9]. Intra-country terroirs have been identified in Brazil and Honduras using sensory scoring methods, with higher altitude terroirs receiving higher scores [1,6]. Descriptive sensory analysis using the varying intensities of turbidity, coffee aroma, green aroma, sweet aroma, acidic taste, bitter taste, and astringent taste identified distinct terroir regions in Paraná, Brazil [5]. Terroir regions were separated by different combinations of aroma descriptors so that no one descriptor defined a region, illustrating the complexity of defining terroir [5].

The primary methods for assessing terroir are the scoring method and the descriptive sensory method. Although the two sensory methods measure related attributes, difficulties have arisen when correlating scoring and sensory descriptive analysis [19]. The results from these two different sensory methods cannot be used interchangeably but could be used in synergy to evaluate the quality of a coffee and its terroir [19]. Defining coffee terroir requires a standard method. The descriptive sensory method allows the coffee taste to be defined rather than its quality. For terroir, the taste carries more weight than the quality, as the quality is dependent on the taster. The recent development of the World Coffee Research Lexicon provides a standard list of sensory attributes for defining terroir by descriptive sensory analysis [20].

### 2.2. How Does the Brew Method Affect the Coffee Terroir Experience?

The coffee brew method has a major effect on the drinker’s sensory experience and can critically affect the assessment of coffee terroir, assuming a consistent roast [15]. There is a growing number of coffee brewing methods used worldwide [41]. The brew method can enhance but not change the sensory attributes of the coffee terroir. The most significant observable difference in sensory attributes is observed with the filter and espresso methods, while the industry standard is the cupping method [18,26,42].

Espresso methods produce a more concentrated coffee and enhance the bitterness of the coffee, favoring roasted and nutty flavor notes [42,43]. The filter method often uses a paper filter, removing oils from the coffee, and can give a less bitter coffee that favors fruity notes [43]. The cupping method sits in the middle, with less intense sensory attributes compared to espresso and retention of the oils compared to the filter method [42,43]. Occupying a middle position means the cupping brew method provides a more centered coffee experience of the terroir.

**Figure 3:**
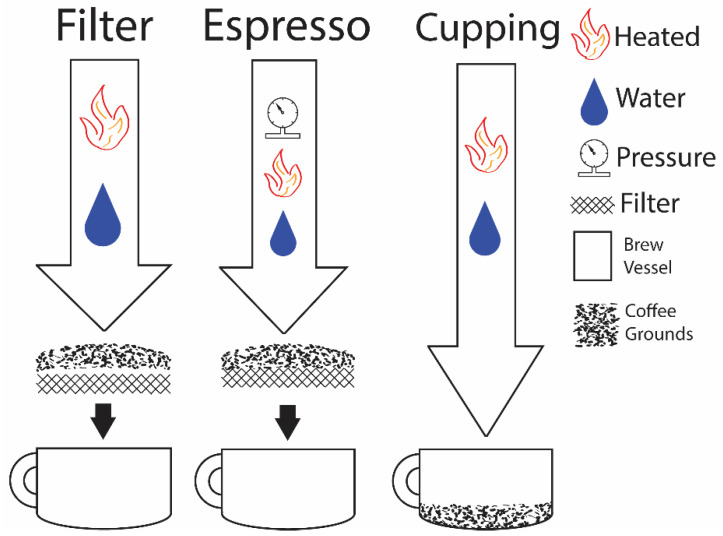
Common literature brew methods presented in a simplified format for easy method comparison.

Coffee brewing is a solid-liquid extraction with water as the solvent. Water temperature and extraction time can significantly affect the variation between coffee brewing methods [15]. The extraction requires a balance between temperature and time, as an increased rate of extraction from higher water temperature can also extract chemicals that can negatively impact the coffee [43]. Filter, espresso, and cupping methods use different parameters and setups for coffee brewing (Table 1, Figure 3), adjusting the sensory experience.

A few studies have successfully used the cupping method to distinguish coffee terroir by countries and regions [5,6,9]. The lack of literature using the more sensory enhancing filter and espresso methods is explained by the robustness of the cupping method (Figure 3) [42]. The cupping method requires minimal specialized equipment and is straightforward in execution, reducing experimental variation and increasing its accessibility [18].

Distinguishing terroir requires a robust, easy to replicate method that the current industry cupping method fulfils [18]. Instead of enhancing the coffee sensory experience, it provides a baseline experience to allow a fair comparison of coffee and its terroir, independent of the brew method. The main limitation of the cupping method is that it does not replicate the consumer experience. However, considering the range of brew methods, it is not plausible to measure terroir using every method [41].

The SCA cupping method requires a coffee to water ratio of 0.055 g/mL (normally, 8.25 g/150 mL), with clean and odor free water poured at 93 °C [18]. Once poured, the coffee steeps for 3–5 min before sensory evaluation [18]. This SCA method also falls within the parameters of the International Organization for Standardization (ISO) method (ISO 6668:2008 Green coffee—Preparation of samples for use in sensory analysis) [26]. The SCA method is a version of the ISO standard targeted toward single-origin coffee. The cupping method focuses on the coffee and the terroir rather than the brewing method, making it the best suited for defining terroir. Using the cupping method, Conley and Wilson [9] identified 36 commonly used sensory descriptors that may be used to distinguish terroir from 742 coffees submitted to the Central American Cup of Excellence program.

### 2.3. How Does the Coffee Particle Size Affect the Experience of the Coffee Terroir?

**Figure 4:**
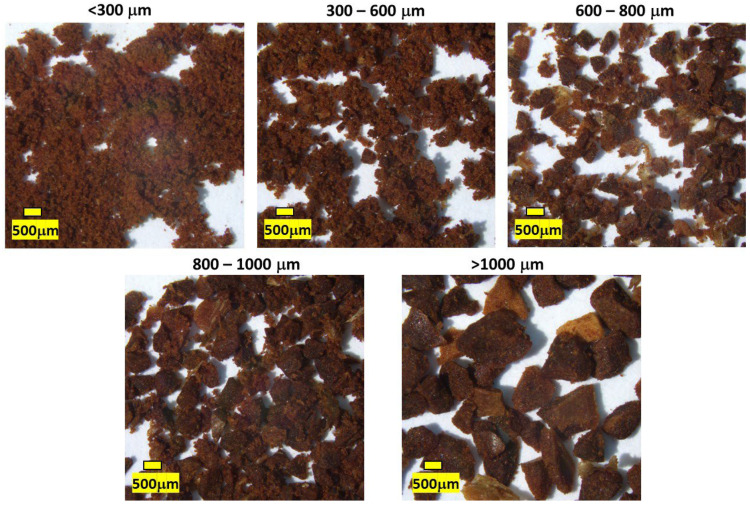
Illustration of ground coffee particle size. A light-medium roasted coffee was ground and separated into size bands using metal sieves.

Coffee beans require grinding to increase the surface area and expose the roasted bean center for brewing [78]. Ground coffee beans range in particle size from around 1800 µm down to <300 µm (Figure 4). Smaller particle size generally increases the intensity of positive and negative sensory attributes due to the greater surface area for extraction [79]. Ideally, the particle size is matched to the brew method to adjust the rate of extraction to enhance the positive and diminish the negative sensory attributes [12].

The ground size is poorly described and inconsistent in the literature [23,56,78,79,80,81], with descriptive definitions used, such as fine, medium, and coarse [79]. Generally, descriptions specify the following: fine ground (300 to 600 μm), medium ground (600 to 800 μm), and coarse ground (800 to >1000 μm) (Figure 4) [23,56,78,79,80,81]. Brew methods utilize different particle sizes, ranging from medium-coarse (Plunger/French Press), medium (Immersion Dripper, Cupping), medium-fine (Pour Over/Filter) and fine (Column/Aeropress) [55,82,83,84,85]. Cupping as an immersive method requires a medium style grind for ideal extraction.

Particle sizes affect the sensory experience, changing the perception of coffee terroir. The flavors of brown roast, burnt wood/ash, cocoa, dark green and hay-like, along with a smoky aroma, are more intense in a fine ground compared to a coarse ground coffee [79]. The SCA cupping method [5,9,86] requires the coffee to be ground so that 70–75% of the grinds pass through a 20 mesh sieve [18], which means 70–75% of the grinds are smaller than 841 μm, about a fine-medium grind [87]. The particle size distribution of ground coffee through a grinder is close to a normal distribution, depending on the grinder and roast used [88].

### 2.4. The Effects of Roasting on the Coffee Terroir Experience

Green coffee beans are roasted in preparation for brewing. The roasting process requires holding the green bean at temperatures of up to 250 °C for a period up to 15 min [89,90]. The longer or hotter the roast, the more Maillard, Strecker, hydrolysis, and pyrolysis reactions can take place, darkening the bean [91]. The roasting process encompasses three phases. The first phase is endothermic, as the moisture in the green bean turns to steam, increasing the internal bean pressure [90]. Once the steam pressure exceeds the cell wall strength, the bean cracks, releasing steam and volatiles while growing in size due to the now weaker cell walls [90]. The second phase is exothermic, with the additional heat from the roaster beginning pyrolysis and Maillard reactions [90]. Aroma formation occurs rapidly during the second phase, which comes to an end with the build-up of carbon dioxide. The remaining steam and volatiles cause a second crack [92]. The third phase after the second crack continues to be exothermic, and the bean begins to burn, creating burnt flavors [92].

**Figure 5:**
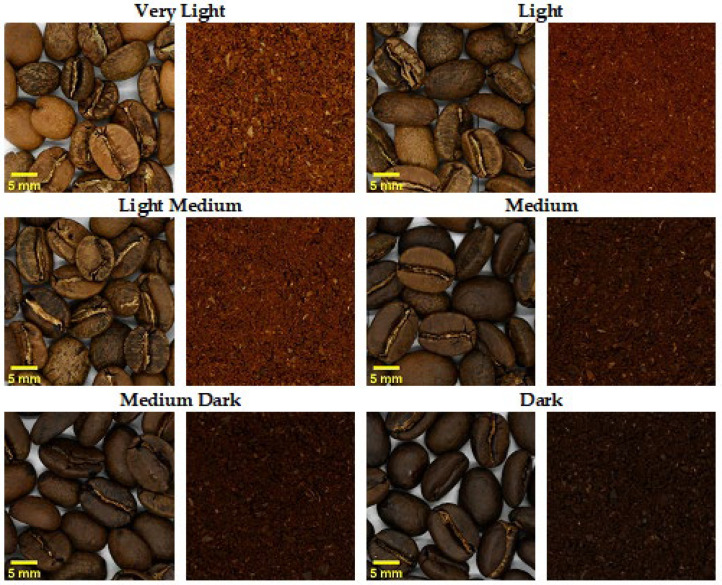
Illustration of coffee bean roast color demonstrating the color of the beans and grounds at different roast levels.

As more prolonged or higher temperature roasts give a darker bean, the color of the roasted bean can indicate the degree of roasting, with the terms ‘light’, ‘medium’, and ‘dark’ used to describe the roast level (Figure 5). Additionally, the bean color correlates well with sensory attributes, allowing the color to indicate the sensory attributes caused by the roasting process [90]. The roasting process causes the roasted bean to increase in volume (50–100%) and lose weight (15–22%) [90]. The change in volume and the degree of roasting influences the uniformity of the grind size [12]. Darker roasts reduce the uniformity of the pores and increase the brittleness of the roasted bean, resulting in a less uniform grind distribution [12]. Lighter roasts provide a more consistent ground size distribution, as the pore sizes remain more uniform [12].

Upon the green beans reaching the roasting step, the terroir is considered fixed, as the roasting process is independent of environmental conditions and location. Further, roasting often occurs in the country of consumption rather than the country of production. When done appropriately, roasting will highlight the characteristics of the terroir, while if carried out inappropriately, it will destroy the characteristics [91]. The coffee terroir determines the starting composition of volatile and non-volatile compounds present during the roasting process, influencing the final aroma and flavor of the coffee [90,91].

The required temperature and time to reach a specific roast color are terroir dependent [1]. Decazy et al. roasted coffee (100 g, 8 min, 220 °C) from 52 plots across six Honduras regions with different terroirs [1]. Under the same roast profile, there was a greater appreciation for coffee beans from the Olancho and El Paraiso regions than those from Santa Barbara [1]. The coffee beans from Santa Barbara exhibited astringent, sour, and acidic flavors, suggesting insufficient roasting [1].

As the terroir governs the roast profile, the color of the roasted ground coffee provides a more consistent means to measure the roast level than the roasting parameters [1]. Terroir-focused studies report the use of medium-light to medium roast levels for cupping [5,6,9], which aligns with the industry standard that requires a medium-light roast (Agtron 65, SCA coffee color scale) [18]. A lighter roast is used, as lighter roasts highlight the differences in post-harvesting methods more than darker roasts [93]. Further, lighter roasts minimize aroma compounds from the roasting process, providing a similar aroma to the green beans (e.g., fruity and sour), and darker roasts increase the aroma compounds created during roasting via Maillard reactions (e.g., bitter, burnt, spicy) [93].

A medium-light roast should be used as the standard roast to ensure that terroir affected by post-harvesting methods can be differentiated through sensory analysis while incorporating reaction products from the roasting process. As coffee roasting often occurs outside the country of origin, it should not contribute to the definition of coffee terroir.

## 3. The Effect of Harvest and Post-Harvest Processing Methods on Terroir

The coffee cherry harvesting method contributes to the terroir and the sensory experience of the coffee. Harvesting of coffee cherries occurs by hand or machine, depending on the terrain and labor cost [94]. Harvesting is complicated by cherries typically not ripening uniformly, with a single branch containing unripe, ripe, and overripe cherries [94]. Hand-picking overcomes the range of ripeness by allowing individual cherries to be picked at optimal ripeness (bright, deep red color) and on any terrain [94]. The downsides to handpicking include increased time required for harvesting and multiple passes through the trees [94]. In contrast, machine harvesting is quicker, using vibrating fingers to knock loose the ripe cherries [94]. Machine harvesting is limited by the terrain and has less selectivity, harvesting a broader range of cherry ripeness, including unripe and overripe cherries in the harvest, dependent on the machine configuration [94].

Post-harvest methods separate the bean from the flesh of the cherry. There are three main methods for separating the bean from the flesh: wash (or wet), honey (or semi-dry), and natural (or dry) methods [14,94]. The wash method removes the flesh mechanically before removing the remaining mucilage using controlled fermentation or mechanical scrubbers. The honey method mechanically de-pulps the cherry before drying with the mucilage remaining on the bean. The wash and honey methods leave the papery parchment layer on the coffee bean. Often, removal of the parchment layer occurs separately to produce the dry green beans used for coffee trading. The natural method generally uses the sun’s heat to dry the cherry, with the dried flesh and mucilage removed in combination with the parchment layer. Each method can be further subdivided, depending on the conditions and additional steps used during post-harvest processing [14]. Each method impacts the sensory experience of the green bean in different ways, with method choice generally dependent on the environmental conditions and resources available. Once processed, the green beans require drying to approximately 11.5% moisture content for all methods before they can be bagged and placed into storage [95].

Selecting ripe cherries on the tree is optimal, as post-harvest sorting will require additional handling steps and create waste. Hand sorting harvested cherries adds another labor-intensive process. Automated color and density sorting can sort ripe cherries from unripe and overripe cherries [94]. Color sorting relies on color sensors to include or exclude cherries based on the cherry color. Density sorting requires flowing water to separate the cherries based on density, assuming similar size, with ripe cherries sinking and less dense unripe and overripe cherries floating. Color sorting can be more effective than density sorting due to variations in cherry size [94].

Historically, post-harvest processing methods arose based on the restrictions of the region where the processing took place. The natural method was suited for regions that were dry and hot during harvesting. In contrast, the wash method allowed processing in regions with high rainfall and humidity, unsuitable for sun drying without harmful fermentation [94]. The natural method generally uses more straightforward equipment than the wash and honey methods, making it more accessible to producers [94]. Colombian coffee is an example of the environment directly affecting the post-harvesting method and terroir [96]. Colombia’s high rainfall and temperature accelerates uncontrolled fermentation in the coffee cherry, preventing the use of natural methods [96]. The limitations imposed by the Colombian environment encourage the use of controlled fermentation (time, temperature, water exchange, and control of spontaneous microorganism development) of the wash method [96].

The harvest method affects terroir, as ripe cherries contribute sweet, floral, and fruity sensory notes. In contrast, unripe cherries contribute grassy, green, or astringent notes and overripe cherries contribute fermented, musty, or moldy notes [94]. Environmental microbes can infest the damaged cherries during the harvesting process, imparting sour notes from uncontrolled fermentation as the cherries are processed [91].

The sensory descriptors red wine, coffee blossom, lemon, acidity, body, fresh butter, and dark chocolate differ significantly across beans from the wash, honey, and natural methods [97]. The wash method adds fruity, sweet, floral, caramel, and acidity sensory notes to the coffee [91,96,97]. Junqueira et al. [96] explains the unique sensory profile of wash methods as a result of fermentation-generated compounds diffusing into the green bean, altering their chemical composition and sensory characteristics. The honey method retains some mucilage on the bean during the drying stage and is said to incorporate sensory characteristics of both wash and natural methods [94,98]. The additional mucilage present on the bean from the honey method increases the polysaccharide content, which could contribute to increased sweet and body sensory notes [99]. The natural method imparts unique red wine and strong body notes [97]. However, as the drying rate is environmentally dependent, the natural method does not produce a consistent coffee, with the intensity of sensory notes varying between batches [94].

Processing close to the growing area contributes to terroir, as the drying conditions, fermentation conditions, and yeast strains used in the post-harvest process can be environmentally dependent. Therefore, for coffee cherries processed on the farm, the environment and geography influence the post-harvesting method, contributing to the coffee terroir. However, not all post-harvest methods can contribute to terroir, as the processing of coffee cherries does not always occur in the same area or environment in which the cherries are grown. The correlation of post-harvest processing methods with terroir defining sensory notes reinforces the need to include the post-harvest method in the definition of coffee terroir. In contrast, the post-harvest processing method is not considered part of the terroir within the wine industry because processing is less likely to be affected by environmental constraints.

## 4. Environmental and Management Factors Affecting Coffee Terroir

**Figure 6:**
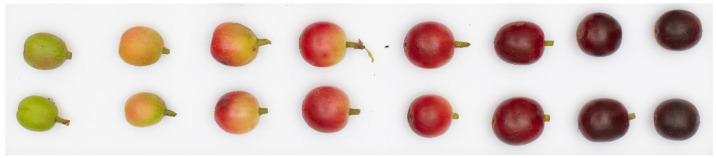
The maturation of arabica coffee cherries from green (unripe) to red (ripe) to overripe (dark red).

Coffee bean quality is maximized by maintaining a lengthy maturation period and ensuring that nutritional and carbon resources required for bean filling are not limited [100]. The maturation rate controls the accumulation rate and ratio of nutrients in the coffee cherry and associated beans [101]. Coffee species differ in their length and rate of the maturation period, with arabica lasting 7–9 months and robusta 11 months, and the cherry changing from green to a red or yellow color (Figure 6) [94]. Each cherry typically contains two coffee beans that accumulate polysaccharides, proteins, lipids, and minerals intended for germination [101]. The cherry forms after pollination, with flowering lasting 2–3 days and requires a period of dryness, followed by 7–10 mm of precipitation to trigger it [101,102]. Flowering can be triggered multiple times throughout the reproductive period during the tropical dry season [101,102]. Multiple flowerings can result in a single branch containing cherries at different stages of maturity, complicating harvesting [101,102].

Ultimately, a trade-off may occur between bean yield per tree and bean size and quality, particularly when resources are limited, because individual beans compete for carbohydrates and nutrients under high bearing loads [103]. As such, negative relationships between coffee bean yields and coffee quality have been reported [104]. Thus, it is the overall interaction between environmental and management factors that affect vegetative growth (‘source’), bean yields (‘sink’), and the length of the maturation period that determine the potential bean quality in a given location and therefore the terroir [105]. The key environmental and agronomic (management) factors that interact to determine coffee bean quality and terroir are discussed below, noting that the interactive effects of these variables can impact differently the different coffee varieties, i.e., genotype x environment effects [106].

### 4.1. Temperatures—Altitude and Shading

Cooler air temperatures during the ripening phase extend the maturation period, and thus coffees grown at high altitude tend to have high bean quality [1,107,108] and are associated with improved sensory experience scores for the coffee beverage [86,109]. Even small changes in temperature of 2.5 °C have a significant effect on the sensory score, changing the quality grade of the coffee and suggesting the possible effect of microclimates on the terroir [109]. Warmer temperature encourages the bioaccumulation of compounds known to contribute to negative green and earthy sensory notes and an increase in compounds that contribute bitter notes in coffee [106,110]. Lower temperatures contribute to fruity notes and acidity [91,110]. Cheng et al. [106] proposed that the increased caffeine content found at higher altitudes corresponds to the more extended maturation period, providing additional time for caffeine accumulation during early bean development. However, this high-caffeine-content coffee grown at high altitudes may be less bitter, as the accumulation of other compounds in a slow maturation could dilute the bitterness [104]. While there are interactive effects of altitude and management factors, including shading and varietal choice, on coffee bean quality (see below), it is generally accepted that higher altitudes are associated with increased bean and cupping quality.

Shading reduces both the air temperature and the radiation reaching the coffee trees. Shading of trees has been widely reported to improve bean quality, particularly at low altitudes, where the cooling effect of shade trees lengthens the bean maturation period [103,111,112]. However, Bosselmann et al. [108] reported a negative association between shading and bean quality, particularly at higher altitudes. Thus, at high altitudes where the maturation period is already lengthened by cooler temperature, any further cooling effects of shade trees may be outweighed by the reduction in radiation reaching the coffee trees. For robusta coffee, a meta-analysis by Piato et al. (2020) found that shade >30% reduced cupping quality, but there were significant interactions with shade and location, variety, rainfall, and tree age; shade had little or negative impacts on trees <16 years old but had positive impacts on older trees [113]. The impact of shading in a particular environment is also influenced by the coffee variety grown [112], and shade trees also impact water use and the dynamics of pathogens and insects, which may also affect coffee tree growth and yields in specific environments [114]. Taken together, these studies highlight that it is the interaction between environmental and agronomic (management) factors that determines bean quality, as opposed to one single factor.

Low temperatures and frost can be detrimental to coffee trees, with frost potential increasing at high altitudes >2000 m in equatorial zones and at low altitudes at higher latitudes [115]. Frost events can cause the death of plants, and low temperatures can affect plant growth, which impacts source–sink relationships. As such, low-temperature stress can affect bean yield and quality, but as an indirect effect it does not affect terroir directly, and thus it is not discussed further in this review.

### 4.2. Moisture Relations—Rainfall and Irrigation

Rainfall affects source–sink relationships by ensuring photosynthesis (carbohydrate source) is not impaired by lack of moisture but also effects time/synchrony of flowering or fruit load by dislodging flowers or immature beans (carbohydrate sink). [116]. A dry period is required to initiate buds prior to flowering, followed by rainfall (or irrigation—see below) to stimulate flowering [117]. The lack of a distinct dry period followed by rain can cause multiple or staggered flowering events that lead to a lack of uniformity in bean maturity on trees. More staggered ripening increases the risk of introducing negative notes by harvesting unripe/overripe cherries in mechanized systems [91,107]. Excessive rainfall during the bean ripening period can also lead to disease issues or bean discoloration, which lowers bean quality by imparting sensory attributes that are considered undesirable [118].

Because moisture impacts both source and sink resources, rainfall can have negative or positive effects on coffee bean quality depending on the timing and amount of precipitation. For example, Decazy et al. (2003) [1] reported higher coffee quality for arabica in regions of Honduras receiving less than 1600 mm rain per annum. Kath et al. (2021) [116] reported an increased risk of small bean size in robusta coffee if <1600 mm rainfall was received during the latter part of the growing season. It is perhaps because of these complexities that some studies have found limited contribution of rainfall or irrigation to coffee terroir [105,119], but the findings need to be taken in context. As an example, irrigation was found to have little influence on the chemical composition and physical quality of beans in several regions of Brazil compared to other site factors [109]. However, it was noted that there were few differences between irrigated and non-irrigated coffee trees in the years of study [109], and hence, one would not expect any impact of irrigation on bean quality. In contrast, in specific environments, irrigation can be used to avoid moisture deficits and maintain bean yields or to initiate flowering to improve uniformity of bean ripening [115,117]. In these scenarios, irrigation will impact bean yields and quality and will thus have an impact on the terroir. Taken together, the results highlight the difficulty in drawing broad conclusions on the role of rainfall and irrigation in terroir when the results from individual studies are generally environment- and season-specific.

### 4.3. Species and Varietal Effects

Arabica (*Coffea arabica*) and robusta (a variety or a hybrid of *C. canephora*) are the main coffees used for commercial production [92,120]. Arabica coffee, by popular opinion, delivers a better sensory experience with characterful, well-defined flavors [92]. Robusta has a less popular sensory experience but contains higher levels of caffeine [92]. Hundreds of varieties exist for the two species, with the more popular arabica having 53 standout varieties recognized by World Coffee Research [121].

The coffee species and variety planted depend on the growing region’s environmental conditions [120]. Arabica and robusta perform best under different growing conditions (Table 2) [91]. Therefore, as species selection is dependent on the place, it forms part of the terroir.

Coffee species and variety selection impact both the plant growth and resulting sensory profile [91,120]. Coffee breeding programs have expanded the selection of coffee varieties that farmers can access [120]. Breeding of arabica has created varieties with increased disease resistance, crop yield, compact growth, and excellent cup quality [120]. Breeding of the naturally more disease-resistant robusta has increased yields and bean size [120]. Coffee traits like bean size, bean weight, fat content, caffeine, and overall liquor standards are highly heritable, providing starting points for the terroir to build on [120]. Though the industry considers arabica to provide a better sensory experience, robusta is more robust and provides greater yields [92].

Variety was found the next most influencing factor on the coffee’s sensory experience after altitude by Aguilar et al. (2012) [119]. For arabica plants grown above 800 m, the Caturra cultivar gives more fruity, acidic, and tarty coffee, while the Typica cultivar gives more balanced and bitter, full-bodied coffee [119]. A study across 10 robusta coffee cultivars planted across five environments in the Amazon region of Brazil identified consistent differences between the sensory notes of varieties, independent of the environment [122]. Sensory notes of chocolate, cereal, woody, herbal, almond, and caramel differentiated the varieties [122]. The species and variety provide the base for building the terroir and determine the plants’ growth and yield under the terroir-defining environmental factors.

### 4.4. Soil and Fertility Management

Soil type generally has a minor impact on terroir compared to other factors [16,119,123], but given that soil type will affect moisture relations and nutrient availability, it has the capacity to influence tree growth and resource availability for bean development. However, both water and nutritional constraints can be overcome by the use of irrigation and fertilizers, depending on availability. Sandy soil was reported to increase bitter notes while reducing fruitiness in coffee in one study [119], which may be related to either soil moisture relations or nutrition, which both affect plant growth. Other than using soil amendments or fertilizers to overcome nutritional constraints to tree growth, there are few reports on the specific impact of soil type or tree nutrition on bean quality, and there is little consensus among the published reports.

Abebe et al. [124] found relationships between cupping quality of Ethiopian coffee and several soil nutrient ratios; for example, Mg:K, P:N, and P:C ratios were all positively associated with cupping quality. However, the notion that specific cation ratios are required for optimal plant growth has been largely discredited [125], and in the absence of any other studies linking soil nutrient ratios to coffee cupping quality, it is difficult to extrapolate the results of Abebe et al. (2019) [124] to draw broader conclusions.

Low potassium resulted in a 10% increase in leaf caffeine concentration in 7-month-old coffee seedlings [126], but any consequence of this finding for bean quality remains unknown. Clemente et al. (2015) reported that a N:K fertilizer ratio of 1:1.56 in hydroponic culture resulted in the greatest cup quality (higher caffeine, color index and sugars, and lower titratable acidity and EC), but the results were based on only four applied K levels [127]. Vinecky et al. (2017) reported increased caffeine concentration with increasing N nutrition under well-watered conditions [128], while Bote and Vos (2021) found increased N nutrition improved bean size and organoleptic qualities under reduced radiation levels [100]. Ultimately, the effect of the addition of any particular nutrient on the bean quality of soil-grown plants will depend on the existing levels of that nutrient and other nutrients within the soil reservoir and on interactions with other factors that drive plant growth, including radiation, temperature, and moisture availability. It is perhaps for this reason that there is no consensus in the literature on the specific impacts of tree nutrition on coffee bean quality, and there are reports of limited effect of soils and fertilizer use on terroir [16,119,123].

### 4.5. Other Environmental and Management Factors Affecting Terroir

Other inherent aspects of the growing site, such as aspect and slope, which impact exposure to sunlight and movement of air, can affect bean quality. For example, Avelino et al. (2005) found higher bean quality from trees on east-facing slopes and attributed this to exposure to more morning sunlight [107]. It is likely, however, that any effect of aspect or slope will interact with other environmental and climatic factors to determine bean quality, and any conclusions on the optimum slope or aspect for coffee production would therefore be location-specific.

Pruning and thinning are management factors that affect source–sink relationships and can therefore impact carbohydrate and nutrient supply during bean maturation. High bearing loads can increase competition between developing beans for carbohydrates, and both pruning and thinning may reduce this competition by minimizing the fruit load (sink) [103]. Pruning typically reduces fruit load in the subsequent season, or seasons, and the impacts on both yield and quality of beans is variety-dependent [129]. Any impact of pruning or thinning will depend heavily on interactions with other environmental variables that affect source–sink relationships in a given season; thus, drawing broad conclusions on the effect of thinning and pruning on terroir is difficult.

## 5. Biochemical Markers and Terroir

A number of biochemical markers have been identified in coffee with different sensory profiles [1,130]. Modern analytical instruments such as gas chromatography mass spectrometry (GCMS) [28,131,132,133,134,135], GCMS-Time of Flight (GCMS-TOF) [136], high-performance liquid chromatography (HPLC) with evaporative light scattering detector (ELSD) [133], near-infrared radiation (NIR) [137], and Raman spectroscopy [138] have been utilized to find relationships between biochemical markers and the sensory notes of coffee.

Using GCMS, robusta and arabica samples from different locations were separated through chemical and sensory notes [135]. Arabica samples presenting acidic, flowery, and fruity notes contained increased amounts of furan derivatives, esters, and ketones. In contrast, robusta samples, with roasty, tobacco, nutty, spicy, and woody notes, had increased amounts of pyrazines and phenolic derivatives [135].

An increase in altitude correlates with an observed increase in chlorogenic acid (1000–1750 cm^−1^) and lipids (2700–3050 cm^−1^) by Raman spectroscopy [138]. The increase in altitude affects the biochemicals of the green bean, with a reported decrease in chocolate and almond notes and an increase in citric, floral, and sugar cane notes [138]. The inclusion of immature beans during harvesting leads to increased levels of serine, aminobutyric acid, valine, leucine, isoleucine, methionine, and 2-methylbutanal, contributing to increased astringency and bitterness in the coffee [131].

Biochemical markers can further separate the country of origin. Naturally processed Uganda robusta reportedly possesses higher acidity from 2,3-butandione and 2,3-pentandione, acetoxy acetone, hexanal, acetic acid, 1-hydroxy-2-butanone, and 1-H-pyrrole-2-carboxaldehyde, which contribute to musty, sour, pungent, and buttery notes [135]. In contrast, woody notes dominate in naturally processed Indonesia robusta, with higher pyrazines and phenolic compounds that contribute woody, spicy, and buttery notes [135]. Ethiopian natural and Papua New Guinea washed arabica present similar differentiation. Ethiopian arabica has a more pungent aroma and fruity notes corresponding to higher levels of furfuryl alcohol, methyl acetate, 5-methyl furfural, and 2-cyclopenten-1-one-3 methyl [135]. In comparison, Papua New Guinea arabica contained higher levels of acetyl furan, 2-furfuryl-5-methylfurane, 2-furanmethanol propanoate, and 2-furfuryl furan, adding sweet-caramel-like notes [135]. Xu et al. (2019) demonstrated that HPLC-quadrupole time-of-flight mass spectrometry (HPLC-QTOF) can identify a number of chemical markers and separate different coffee origins (Ethiopia, Colombia, and China) [15]. However, additional sensory analysis is required to link the chemical markers with terroir [15].

**Figure 7:**
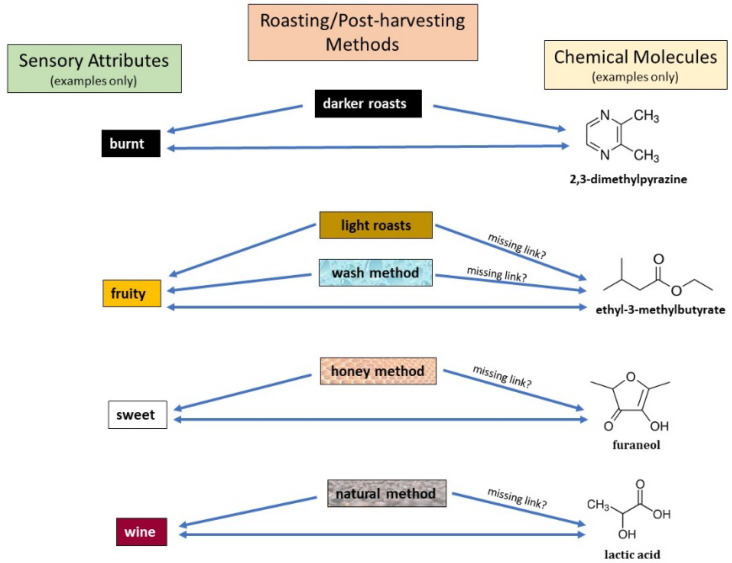
Associations between sensory attributes, molecules, and different coffee roasting/post-harvesting methods [10,141].

Biochemical markers allow separation based on the presence and absence of compounds and the variations in the quantities present. A recent review by Seninde et al. goes deeper into the relationship between biomarkers and sensory notes [10]. As the reproduction of sensory analysis heavily relies on the panelists selected each time, biochemical markers and instrumental analysis should be used to assist in identifying terroir and quality control. Some examples have been provided in Figure 7 to assist in the understanding of the relationship between sensory attribute, chemical molecules, and different coffee roasting/post-harvesting methods. Some links between the chemical molecules and roasting or post-harvesting methods are still not clear and sometimes are even contradictory in publications [10,139,140,141].

## 6. Conclusions

Coffee terroir is greatly affected by environmental and agricultural factors that affect the maturation rate of the coffee cherry and bean. The remaining factors like soil and water affect the nutrients available to the plant and coffee cherry development. Plant nutrition to terroir correlations are inconclusive. In contrast to wine, the post-harvest processing of the coffee cherry can be considered part of the terroir, as environmental factors often determine how the cherry is processed.

We propose that coffee terroir should be assessed using descriptive sensory analysis using the cupping method for a medium-light roast. The post-harvesting method, coffee variety, and environmental and farm management factors should all be considered as contributors to the coffee terroir.

The assessment of terroir through the cupping method represents the industry standard but does not represent the consumer experience. After the terroir is assessed by the cupping method, different roast profiles and brew methods should be tested to check if the terroir could be retained or highlighted. Modern instrumental analysis can identify biochemical markers associated with terroir and should be used to validate the sensory assessment from a cupping panel.

## Figures and Tables

**Table 1:** A summary of the brew parameters presented in the literature for cupping, filter, and espresso methods.

Brew Method	Cupping ^1^	Filter ^2^	Espresso ^3^
Coffee Grounds (g)	5–8.25	2–210	5.5–21
Water (mL)	100–300	100–3800	20–100
Water Temperature (°C)	92—Boiling	90–100	86–97
Brew Time (min)	3–5	2–10	10–30 (s)
Pressure (bar)	NA	NA	8.5–19
Water/Coffee ratio	14.29–20.00	7.4–59.0	2–18.18
Filter Type	NA	Paper Filter #3, Paper Filter #6, Paper Filter, Metallic Sieve, Ceramic Filter, Metal Filter, Stainless Steel Tea Strainer	Metallic Sieve

^1^ [18,26,34,42,44,45,46,47,48,49,50,51], ^2^ [22,27,39,51,52,53,54,55,56,57,58,59,60,61,62,63,64,65,66], ^3^ [38,59,60,61,67,68,69,70,71,72,73,74,75,76,77].

**Table 2:** Ideal growing conditions for arabica and robusta coffee species [91].

	Arabica	Robusta
Altitude (m)	1000–2100	100–1000
Daily Ave Temp (°C)	18–22	22–26
Annual Rainfall (mm)	1500–2500	>2000
Sunlight	Partial Shade	Full Sun

## Data Availability

No new data were created or analyzed in this study. Data sharing is not applicable to this article.

## References

[1] Decazy F., Avelino J., Guyot B., Perriot J.J., Pineda C., Cilas C. (2003). Quality of different Honduran coffees in relation to several environments. J. Food Sci..

[2] Vaudour E., Costantini E., Jones G.V., Mocali S. (2015). An overview of the recent approaches to terroir functional modelling, footprinting and zoning. Soil.

[3] Anesi A., Stocchero M., Dal Santo S., Commisso M., Zenoni S., Ceoldo S., Tornielli G.B., Siebert T.E., Herderich M., Pezzotti M. (2015). Towards a scientific interpretation of the terroir concept: Plasticity of the grape berry metabolome. BMC Plant Biol..

[4] Van Leeuwen C., Barbe J.-C., Darriet P., Geffroy O., Gomes E., Guillaumie S., Helwi P., Laboyrie J., Lytra G., Le Menn N. (2020). Recent advancements in understanding the terroir effect on aromas in grapes and wines. Oeno One.

[5] Scholz M., Kitzberger C.S.G., Prudencio S.H., Silva R. (2018). The typicity of coffees from different terroirs determined by groups of physico-chemical and sensory variables and multiple factor analysis. Food Res. Int..

[6] Silva S.D.A., de Queiroz D.M., Ferreira W.P., Correa P.C., Rufino J.L. (2016). Mapping the potential beverage quality of coffee produced in the Zona da Mata, Minas Gerais, Brazil. J. Sci. Food Agric..

[7] International Organisation of Vine and Wine (2010). Definition of Vitivinicultural “Terroir”. Resolution OIV/VITI 333/2010.

[8] Souza Gonzaga L., Capone D.L., Bastian S.E.P., Jeffery D.W. (2021). Defining wine typicity: Sensory characterisation and consumer perspectives. Aust. J. Grape Wine Res..

[9] Conley J., Wilson B. (2020). Coffee terroir: Cupping description profiles and their impact upon prices in Central American coffees. GeoJournal.

[10] Seninde D.R., Chambers E. (2020). Coffee Flavor: A Review. Beverages.

[11] Hu G.L., Peng X.R., Gao Y., Huang Y.J., Li X., Su H.G., Qiu M.H. (2020). Effect of roasting degree of coffee beans on sensory evaluation: Research from the perspective of major chemical ingredients. Food Chem..

[12] Cordoba N., Fernandez-Alduenda M., Moreno F.L., Ruiz Y. (2020). Coffee extraction: A review of parameters and their influence on the physicochemical characteristics and flavour of coffee brews. Trends Food Sci. Technol..

[13] Haile M., Kang W.H. (2019). The Role of Microbes in Coffee Fermentation and Their Impact on Coffee Quality. J. Food Qual..

[14] Hameed A., Hussain S.A., Ijaz M.U., Ullah S., Pasha I., Suleria H.A.R. (2018). Farm to Consumer: Factors Affecting the Organoleptic Characteristics of Coffee. II: Postharvest Processing Factors. Compr. Rev. Food. Sci. Food Saf..

[15] Xu L., Lao F., Xu Z., Wang X., Chen F., Liao X., Chen A., Yang S. (2019). Use of liquid chromatography quadrupole time-of-flight mass spectrometry and metabolomic approach to discriminate coffee brewed by different methods. Food Chem..

[16] Lambot C., Herrera J.C., Bertrand B., Sadeghian S., Benavides P., Gaitán A. (2017). Cultivating coffee quality-terroir and agro-Ecosystem. The Craft and Science of Coffee.

[17] Thomas E., Puget S., Valentin D., Songer P. (2017). Sensory evaluation—Profiling and preferences. The Craft and Science of Coffee.

[18] Specialty Coffee Association (2018). Coffee Standards.

[19] Di Donfrancesco B., Guzman N.G., Chambers E. (2014). Comparison of results from cupping and descriptive sensory analysis of Colombian brewed coffee. J. Sens. Stud..

[20] Sensory Lexicon Advisory Group (2017). World Coffee Research Sensory Lexicon.

[21] Steen I., Waehrens S.S., Petersen M.A., Munchow M., Bredie W.L. (2017). Influence of serving temperature on flavour perception and release of Bourbon Caturra coffee. Food Chem..

[22] Barahona I., Sanmiguel Jaimes E.M., Yang J.B. (2020). Sensory attributes of coffee beverages and their relation to price and package information: A case study of Colombian customers’ preferences. Food Sci. Nutr..

[23] Cordoba N., Pataquiva L., Osorio C., Moreno F.L.M., Ruiz R.Y. (2019). Effect of grinding, extraction time and type of coffee on the physicochemical and flavour characteristics of cold brew coffee. Sci. Rep..

[24] Angeloni G., Guerrini L., Masella P., Innocenti M., Bellumori M., Parenti A. (2019). Characterization and comparison of cold brew and cold drip coffee extraction methods. J. Sci. Food Agric..

[25] (2018). Coffee—Sensory Analysis—Vocabulary.

[26] (2017). Green Coffee—Preparation of Samples for Use in Sensory Analysis.

[27] Bressanello D., Liberto E., Cordero C., Rubiolo P., Pellegrino G., Ruosi M.R., Bicchi C. (2017). Coffee aroma: Chemometric comparison of the chemical information provided by three different samplings combined with GC-MS to describe the sensory properties in cup. Food Chem..

[28] Bolivar J.T.C., Pérez W.R., Salazar J.C.S., Espinosa C.M.O., Cano G.A.V. (2017). Minority compounds and sensory analysis evaluation of *Coffea arabica* var. caturra cultivated in three different altitudinal ranges. Acta Agron..

[29] Cafe Imports Department of Sensory Analysis (2017). Analytic Cupping Score Card.

[30] Sunarharum W.B. (2016). The Compositional Basis of Coffee Flavour. Ph.D. Thesis.

[31] Silveira A.S., Pinheiro A.C.T., Ferreira W.P.M., Silva L.J., Rufino J.L.S., Sakiyama N.S. (2016). Sensory analysis of specialty coffee from different environmental conditions in the region of matas de minas, minas gerais, Brazil. Rev. Ceres.

[32] Chambers E., Sanchez K., Phan U.X.T., Miller R., Civille G.V., Di Donfrancesco B. (2016). Development of a “living” lexicon for descriptive sensory analysis of brewed coffee. J. Sens. Stud..

[33] Evangelista S.R., Silva C.F., Miguel M., Cordeiro C.D., Pinheiro A.C.M., Duarte W.F., Schwan R.F. (2014). Improvement of coffee beverage quality by using selected yeasts strains during the fermentation in dry process. Food Res. Int..

[34] Hetzel A. (2011). Fine Robusta Standards and Protocols v1.1.

[35] Borsato D., Pina M.V.R., Spacino K.R., Scholz M.B.D., Androcioli Filho A. (2011). Application of artificial neural networks in the geographical identification of coffee samples. Eur. Food Res. Technol..

[36] Pérez-Martínez M., Sopelana P., De Peña M.P., Cid C. (2008). Effects of refrigeration and oxygen on the coffee brew composition. Eur. Food Res. Technol..

[37] Brazilian Coffee Industry Association (2004). Recommended Quality Standard and Best Practices Manufacture of Roasted Grain Coffee and Roasted and Ground Coffee.

[38] Maeztu L., Andueza S., Ibanez C., de Pena M.P., Bello J., Cid C. (2001). Multivariate methods for characterization and classification of espresso coffees from different botanical varieties and types of roast by foam, taste, and mouthfeel. J. Agric. Food Chem..

[39] Grupo de Avaliação do Café (2000). FO-055 GAC Report Model.

[40] Alliance for Coffee Excellence (2002). Cup of Excellence Forms.

[41] Caporaso N., Genovese A., Canela M.D., Civitella A., Sacchi R. (2014). Neapolitan coffee brew chemical analysis in comparison to espresso, moka and American brews. Food Res. Int..

[42] Sanchez K., Chambers E. (2015). How does product preparation affect sensory properties? An example with coffee. J. Sens. Stud..

[43] Gloess A.N., Schönbächler B., Klopprogge B., D’Ambrosio L., Chatelain K., Bongartz A., Strittmatter A., Rast M., Yeretzian C. (2013). Comparison of nine common coffee extraction methods: Instrumental and sensory analysis. Eur. Food Res. Technol..

[44] Nebesny E., Budryn G. (2006). Evaluation of sensory attributes of coffee brews from robusta coffee roasted under different conditions. Eur. Food Res. Technol..

[45] Marin K., Požrl T., Zlatić E., Plestenjak A. (2008). A New Aroma Index to Determine the Aroma Quality of Roasted and Ground Coffee During Storage. Food Technol. Biotechnol..

[46] Specialty Coffee Association of America (2015). SCAA Standard. Golden Cup.

[47] Lingle T.R., Menon S.N. (2017). Cupping and Grading—Discovering Character and Quality. The Craft and Science of Coffee.

[48] Pereira L.L., Cardoso W.S., Guarconi R.C., da Fonseca A.F.A., Moreira T.R., ten Caten C.S. (2017). The consistency in the sensory analysis of coffees using Q-graders. Eur. Food Res. Technol..

[49] Pereira L.L., Guarconi R.C., de Souza G.S., Brioschi D., Moreira T.R., ten Caten C.S. (2018). Propositions on the Optimal Number of Q-Graders and R-Graders. J. Food Qual..

[50] Kulapichitr F., Borompichaichartkul C., Suppavorasatit I., Cadwallader K.R. (2019). Impact of drying process on chemical composition and key aroma components of Arabica coffee. Food Chem..

[51] WCE Rules and Regulations Committee (2020). 2020 World Cup Tasters Championship Official Rules and Regulations.

[52] Abrahao S.A., Pereira R., de Sousa R.V., Lima A.R., Crema G.P., Barros B.S. (2013). Influence of Coffee Brew in Metabolic Syndrome and Type 2 Diabetes. Plant Food Hum. Nutr..

[53] Batali M.E., Frost S.C., Lebrilla C.B., Ristenpart W.D., Guinard J.X. (2020). Sensory and monosaccharide analysis of drip brew coffee fractions versus brewing time. J. Sci. Food Agric..

[54] Cammerer B., Kroh L.W. (2006). Antioxidant activity of coffee brews. Eur. Food Res. Technol..

[55] Technical Standards Committee (2016). Guidelines for Brewing with a Two Cup Pour-Over Brewer. SCAA Best Practice.

[56] Fibrianto K., Febryana Y.R., Wulandari E.S. (2018). Effect of brewing technique and particle size of the ground coffee on sensory profiling of brewed Dampit robusta coffee. Proceedings of the International Conference on Green Agro-Industry and Bioeconomy.

[57] Fujioka K., Shibamoto T. (2008). Chlorogenic acid and caffeine contents in various commercial brewed coffees. Food Chem..

[58] Han J., Kim M.K., Lee K.G. (2017). Furan Levels and Sensory Profiles of Commercial Coffee Products Under Various Handling Conditions. J. Food Sci..

[59] López-Galilea I., Fournier N., Cid C., Guichard E. (2006). Changes in headspace volatile concentrations of coffee brews caused by the roasting process and the brewing procedure. J. Agric. Food Chem..

[60] Ludwig I.A., Sanchez L., Caemmerer B., Kroh L.W., De Pena M.P., Cid C. (2012). Extraction of coffee antioxidants: Impact of brewing time and method. Food Res. Int..

[61] Niseteo T., Komes D., Belščak-Cvitanović A., Horžić D., Budeč M. (2012). Bioactive composition and antioxidant potential of different commonly consumed coffee brews affected by their preparation technique and milk addition. Food Chem..

[62] Rendón M.Y., De Jesus Garcia Salva T., Bragagnolo N. (2014). Impact of chemical changes on the sensory characteristics of coffee beans during storage. Food Chem..

[63] Seo H.S., Lee M., Jung Y.J., Hwang I. (2009). A novel method of descriptive analysis on hot brewed coffee: Time scanning descriptive analysis. Eur. Food Res. Technol..

[64] Scholz M.B.D.S., Silva J.V.N.D., Figueiredo V.R.G.D., Kitzberger C.S.G. (2013). Sensory atributes and physico-chemical characteristics of the coffee beverage from the IAPAR cultivars. Coffee Sci..

[65] Sittipod S., Schwartz E., Paravisini L., Peterson D.G. (2019). Identification of flavor modulating compounds that positively impact coffee quality. Food Chem..

[66] Tfouni S.A.V., Serrate C.S., Leme F.M., Camargo M.C.R., Teles C.R.A., Cipolli K., Furlani R.P.Z. (2013). Polycyclic aromatic hydrocarbons in coffee brew: Influence of roasting and brewing procedures in two Coffea cultivars. LWT Food Sci. Technol..

[67] Alves R.C., Almeida I.M.C., Casal S., Oliveira M. (2010). Isoflavones in Coffee: Influence of Species, Roast Degree, and Brewing Method. J. Agric. Food Chem..

[68] Golden Bean Australia Competition entry guidelines golden bean coffee roasters competition and conference. Proceedings of the Golden Bean Coffee Roasters Competition and Conference.

[69] WCE Rules and Regulations Committee (2019). 2020 World Barista Championship Rules and Regulations.

[70] Kim S.Y., Kang B.S. (2018). A colorimetric sensor array-based classification of coffees. Sens. Actuators B Chem..

[71] Royal Queensland Show (2020). 2020 Royal Queensland Coffee Awards.

[72] Maeztu L., Sanz C., Andueza S., De Pena M.P., Bello J., Cid C. (2001). Characterization of espresso coffee aroma by static headspace GC-MS and sensory flavor profile. J. Agric. Food Chem..

[73] Navarini L., Ferrari M., Liverani F.S., Liggieri L., Ravera F. (2004). Dynamic tensiometric characterization of espresso coffee beverage. Food Hydrocoll..

[74] Fadhil R., Nurba D. (2019). Comparison of Gayo Arabica coffee taste sensory scoring system between Eckenrode and Fuzzy-Eckenrode methods. IOP Conf. Ser. Earth Environ. Sci..

[75] Pérez-Martínez M., Caemmerer B., De Peña M.P., Concepción C., Kroh L.W. (2010). Influence of brewing method and acidity regulators on the antioxidant capacity of coffee brews. J. Agric. Food Chem..

[76] Parenti A., Guerrini L., Masella P., Spinelli S., Calamai L., Spugnoli P. (2014). Comparison of espresso coffee brewing techniques. J. Food Eng..

[77] Alves R.C., Soares C., Casal S., Fernandes J.O., Oliveira M. (2010). Acrylamide in espresso coffee: Influence of species, roast degree and brew length. Food Chem..

[78] Bell L.N., Wetzel C.R., Grand A.N. (1996). Caffeine content in coffee as influenced by grinding and brewing techniques. Food Res. Int..

[79] Frost S.C., Ristenpart W.D., Guinard J.X. (2019). Effect of Basket Geometry on the Sensory Quality and Consumer Acceptance of Drip Brewed Coffee. J. Food Sci..

[80] Gniechwitz D., Brueckel B., Reichardt N., Blaut M., Steinhart H., Bunzel M. (2007). Coffee dietary fiber contents and structural characteristics as influenced by coffee type and technological and brewing procedures. J. Agric. Food Chem..

[81] Lee S.J., Kim M.K., Lee K.-G. (2017). Effect of reversed coffee grinding and roasting process on physicochemical properties including volatile compound profiles. Innov. Food Sci. Emerg. Technol..

[82] Technical Standards Committee (2016). Guidelines for brewing with a column brewer. SCAA Best Practice.

[83] Technical Standards Committee (2016). Guidelines for brewing with a single cup immersion drippe. SCAA Best Practice.

[84] Technical Standards Committee (2016). Guidelines for brewing with a three cup french press. SCAA Best Practice.

[85] Technical Standards Committee (2015). SCAA Protocols. Cupping Specialty Coffee.

[86] Smith J. (2018). Coffee Landscapes: Specialty Coffee, Terroir, and Traceability in Costa Rica. Cult. Agric. Food Environ..

[87] AZoM (2002). com. Particle Size—US Sieve Series and Tyler Mesh Size Equivalents.

[88] Moeenfard M., Silva J.A., Borges N., Santos A., Alves A. (2015). Diterpenes in espresso coffee: Impact of preparation parameters. Eur. Food Res. Technol..

[89] Laukaleja I., Kruma Z. Influence of the roasting process on bioactive compounds and aroma profile in specialty coffee: A review. Proceedings of the Baltic Conference on Food Science and Technology and North and East European Congress on Food.

[90] Münchow M., Alstrup J., Steen I., Giacalone D. (2020). Roasting Conditions and Coffee Flavor: A Multi-Study Empirical Investigation. Beverages.

[91] Toledo P.R.A.B., Pezza L., Pezza H.R., Toci A.T. (2016). Relationship Between the Different Aspects Related to Coffee Quality and Their Volatile Compounds. Compr. Rev. Food. Sci. Food Saf..

[92] Stephenson T. (2019). The Curious Barista’s Guide to Coffee.

[93] Gonzalez-Rios O., Suarez-Quiroz M.L., Boulanger R., Barel M., Guyot B., Guiraud J.P., Schorr-Galindo S. (2007). Impact of “ecological” post-harvest processing on coffee aroma: II. Roasted coffee. J. Food Compos. Anal..

[94] Batista L.R., de Souza S.M.C., e Batista C.F.S., Schwan R.F. (2016). Coffee: Types and production. Encyclopedia of Food and Health.

[95] Carmo K.B., Carmo J.C.B., Krause M.R., Moreli A.P., Lo Monaco P.A.V. (2020). Quality of arabic coffee under different processing systems, drying methods and alti-tudes. Biosci. J..

[96] Junqueira A.C.D., Pereira G.V.D., Medina J.D.C., Alvear M.C.R., Rosero R., Neto D.P.D., Enriquez H.G., Soccol C.R. (2019). First description of bacterial and fungal communities in Colombian coffee beans fermentation analysed using Illumina-based amplicon sequencing. Sci. Rep..

[97] Partida-Sedas J.G., Ferreiro M.N.M., Vazquez-Oderiz M.L., Romero-Rodriguez M.A., Perez-Portilla E. (2019). Influence of the postharvest processing of the “Garnica” coffee variety on the sensory characteristics and overall acceptance of the beverage. J. Sens. Stud..

[98] Sanz-Uribe J.R., Yusianto, Menon S.N., Peñuela A., Oliveros C., Husson J., Brando C., Rodriguez A. (2017). Postharvest processing—Revealing the green bean. The Craft and Science of Coffee.

[99] Pereira L.L., Guarçoni R.C., Pinheiro P.F., Osório V.M., Pinheiro C.A., Moreira T.R., ten Caten C.S. (2020). New propositions about coffee wet processing: Chemical and sensory perspectives. Food Chem..

[100] Bote A.D., Vos J. (2021). Tree management and environmental conditions affect coffee (*Coffea arabica* L.) bean quality. NJAS Wagening. J. Life Sci..

[101] Eira M.T.S., Silva E.A., De Castro R.D., Dussert S., Walters C., Bewley J.D., Hilhorst H.W.M. (2006). Coffee seed physiology. Braz. J. Plant Physiol..

[102] Schmitt L., Perfecto I. (2020). Who gives a flux? Synchronous flowering of *Coffea arabica* accelerates leaf litter decomposition. Ecosphere.

[103] Vaast P., Bertrand B., Perriot J.-J., Guyot B., Génard M. (2006). Fruit thinning and shade improve bean characteristics and beverage quality of coffee (*Coffea arabica* L.) under optimal conditions. J. Sci. Food Agric..

[104] Avelino J., Perriot J.J., Guyot B., Pineda C., Decazy F., Cilas C. (2002). Identifying Terroir Coffees in Honduras. Research and Coffee Growing.

[105] Oberthur T., Laderach P., Posada H., Fisher M.J., Samper L.F., Illera J., Collet L., Moreno E., Alarcon R., Villegas A. (2011). Regional relationships between inherent coffee quality and growing environment for denomination of origin labels in Narino and Cauca, Colombia. Food Policy.

[106] Cheng B., Furtado A., Smyth H.E., Henry R.J. (2016). Influence of genotype and environment on coffee quality. Trends Food Sci. Technol..

[107] Avelino J., Barboza B., Araya J.C., Fonseca C., Davrieux F., Guyot B., Cilas C. (2005). Effects of slope exposure, altitude and yield on coffee quality in two altitude terroirs of Costa Rica, Orosi and Santa Maria de Dota. J. Sci. Food Agric..

[108] Bosselmann A.S., Dons K., Oberthur T., Olsen C.S., Raebild A., Usma H. (2009). The influence of shade trees on coffee quality in small holder coffee agroforestry systems in Southern Colombia. Agric. Ecosyst. Environ..

[109] Da Silva E.A., Mazzafera P., Brunini O., Sakai E., Arruda F.B., Mattoso L.H.C., Carvalho C.R.L., Pires R.C.M. (2005). The influence of water management and environmental conditions on the chemical composition and beverage quality of coffee beans. Braz. J. Plant Physiol..

[110] Bertrand B., Boulanger R., Dussert S., Ribeyre F., Berthiot L., Descroix F., Joët T. (2012). Climatic factors directly impact the volatile organic compound fingerprint in green Arabica coffee bean as well as coffee beverage quality. Food Chem..

[111] Geromel C., Ferreira L.P., Guerreiro S.M., Cavalari A.A., Pot D., Pereira L.F., Leroy T., Vieira L.G., Mazzafera P., Marraccini P. (2006). Biochemical and genomic analysis of sucrose metabolism during coffee (*Coffea arabica*) fruit development. J. Exp. Bot..

[112] Muschler R.G. (2001). Shade improves coffee quality in a sub-optimal coffee-zone of Costa Rica. Agrofor. Syst..

[113] Piato K., Lefort F., Subia C., Caicedo C., Calderon D., Pico J., Norgrove L. (2020). Effects of shade trees on robusta coffee growth, yield and quality. A meta-analysis. Agron. Sustain. Dev..

[114] Beer J. (1987). Advantages, disadvantages and desirable characteristics of shade trees for coffee, cacao and tea. Agrofor. Syst..

[115] DaMatta F.M., Ramalho J.D.C. (2006). Impacts of drought and temperature stress on coffee physiology and production: A review. Braz. J. Plant Physiol..

[116] Kath J., Byrareddy V.M., Mushtaq S., Craparo A., Porcel M. (2021). Temperature and rainfall impacts on robusta coffee bean characteristics. Clim. Risk Manag..

[117] Carr M.K.V. (2001). The water relations and irrigation requirements of coffee. Exp. Agric..

[118] Taniwaki M.H., Teixeira A.A., Teixeira A.R.R., Copetti M.V., Iamanaka B.T. (2014). Ochratoxigenic fungi and ochratoxin A in defective coffee beans. Food Res. Int..

[119] Aguilar P., Ribeyre F., Escarraman A., Bastide P., Berthiot L. (2012). Sensory profiles of coffee in the Dominican Republic are linked to the terroirs. Cah. Agric..

[120] Herrera J.C., Lambot C. (2017). The coffee tree—Genetic diversity and origin. The Craft and Science of Coffee.

[121] World Coffee Research (2018). Arabica Coffee Varieties.

[122] Teixeira A.L., Rocha R.B., Espindula M.C., Ramalho A.R., Vieira J.R., Alves E.A., Lunz A.M.P., Souza F.D., Costa J.N.M., Fernandes C.D. (2020). Amazonian Robustas—New *Coffea canephora* coffee cultivars for the Western Brazilian Amazon. Crop Breed. Appl. Biotechnol..

[123] Morales-Ramos V., Escamilla-Prado E., Ruiz-Carbajal R.A., Perez-Sato J.A., Velazquez-Morales J.A., Servin-Juarez R. (2020). On the soil-bean-cup relationships in *Coffea arabica* L.. J. Sci. Food Agric..

[124] Abebe Y., Juergen B., Endashaw B., Kitessa H., Heiner G. (2019). The role of soil nutrient ratios in coffee quality: Their influence on bean size and cup quality in the natural coffee forest ecosystems of Ethiopia. Afr. J. Agric. Res..

[125] Kopittke P.M., Menzies N.W. (2007). A Review of the Use of the Basic Cation Saturation Ratio and the “Ideal” Soil. Soil Sci. Soc. Am. J..

[126] Mazzafera P. (1999). Mineral nutrition and caffeine content in coffee leaves. Bragantia.

[127] Clemente J.M., Martinez H.E.P., Alves L.C., Finger F.L., Cecon P.R. (2015). Effects of nitrogen and potassium on the chemical composition of coffee beans and on beverage quality. Acta Sci. Agron..

[128] Vinecky F., Davrieux F., Mera A.C., Alves G.S.C., Lavagnini G., Leroy T., Bonnot F., Rocha O.C., Bartholo G.F., Guerra A.F. (2017). Controlled irrigation and nitrogen, phosphorous and potassium fertilization affect the biochemical composition and quality of Arabica coffee beans. J. Agric. Sci..

[129] Nadaleti D.H.S., Vilela D.J.M., Carvalho G.R., de Mendonça J.M.A., Botelho C.E., Coelho L.S., de Oliveira Fassio L., Carvalho J.P.F., Fassio L., de Minas E.P. (2018). Productivity and sensory quality of arábica coffee in response to pruning type “esqueletamento”. J. Agric. Sci..

[130] Wilson B.R., Conley J.F., Harris T.M., Lafone F. (2012). New terrains of taste: Spatial analysis of price premiums for single origin coffees in Central America. Appl. Geogr..

[131] Rabelo M.H.S., Borem F.M., de Lima R.R., Alves A.P.D., Pinheiro A.C.M., Ribeiro D.E., dos Santos C.M., Pereira R. (2021). Impacts of quaker beans over sensory characteristics and volatile composition of specialty natural coffees. Food Chem..

[132] Cain C.N., Haughn N.J., Purcell H.J., Marney L.C., Synovec R.E., Thoumsin C.T., Jackels S.C., Skogerboe K.J. (2021). Analytical Determination of the Severity of Potato Taste Defect in Roasted East African Arabica Coffee. J. Agric. Food Chem..

[133] Wang C.H., Sun J.C., Lassabliere B., Yu B., Liu S.Q. (2020). Coffee flavour modification through controlled fermentation of green coffee beans by *Saccharomyces cerevisiae* and *Pichia kluyveri*: Part II. Mixed cultures with or without lactic acid bacteria. Food Res. Int..

[134] Aditiawati P., Astuti D.I., Kriswantoro J.A., Khanza S.M., Kamarisima, Irifune T., Amalia F., Fukusaki E., Putri S.P. (2020). GC/MS-based metabolic profiling for the evaluation of solid state fermentation to improve quality of Arabica coffee beans. Metabolomics.

[135] Bressanello D., Liberto E., Cordero C., Sgorbini B., Rubiolo P., Pellegrino G., Ruosi M.R., Bicchi C. (2018). Chemometric Modeling of Coffee Sensory Notes through Their Chemical Signatures: Potential and Limits in Defining an Analytical Tool for Quality Control. J. Agric. Food Chem..

[136] Cui D.D., Liu Y., Chen Y.P., Feng X., Lu Y., Yu B. (2020). Application of SPME-GC-TOFMS, E-nose, and sensory evaluation to investigate the flavor characteristics of Chinese Yunnan coffee at three different conditions (beans, ground powder, and brewed coffee). Flavour Fragr. J..

[137] Chang Y.T., Hsueh M.C., Hung S.P., Lu J.M., Peng J.H., Chen S.F. (2021). Prediction of specialty coffee flavors based on near-infrared spectra using machine- and deep-learning methods. J. Sci. Food Agric..

[138] Martins P.M.M., Batista N.N., Miguel M., Simao J.B.P., Soares J.R., Schwan R.F. (2020). Coffee growing altitude influences the microbiota, chemical compounds and the quality of fermented coffees. Food Res. Int..

[139] Laukaleja I., Koppel K. (2021). Aroma active compound perception in differently roasted and brewed coffees by gas chromatography–olfactometry. J. Sens. Stud..

[140] Wang X., Wang Y., Hu G., Hong D., Guo T., Li J., Li Z., Qiu M. (2022). Review on factors affecting coffee volatiles: From seed to cup. J. Sci. Food Agric..

[141] Yang N., Liu C., Liu X., Degn T.K., Munchow M., Fisk I. (2016). Determination of volatile marker compounds of common coffee roast defects. Food Chem..

